# Elevated Depressive Symptoms Shape Gut Barrier Integrity, LPS Translocation, and PUFA Composition in IBS-D: Evidence from a Low-FODMAP Dietary Intervention

**DOI:** 10.3390/nu18091473

**Published:** 2026-05-05

**Authors:** Laura Prospero, Michele Linsalata, Giuseppe Riezzo, Antonella Orlando, Antonia Ignazzi, Benedetta D’Attoma, Domenica Mallardi, Maria Notarnicola, Valeria Tutino, Valentina De Nunzio, Giuliano Pinto, Francesco Russo

**Affiliations:** 1 Functional Gastrointestinal Disorders Research Group, National Institute of Gastroenterology IRCCS “Saverio de Bellis”, 70013 Castellana Grotte, Italy; laura.prospero@irccsdebellis.it (L.P.); michele.linsalata@irccsdebellis.it (M.L.); giuseppe.riezzo@irccsdebellis.it (G.R.); antonella.orlando@irccsdebellis.it (A.O.); antonia.ignazzi@irccsdebellis.it (A.I.); benedetta.dattoma@irccsdebellis.it (B.D.); domenica.mallardi@irccsdebellis.it (D.M.); 2 Laboratory of Nutritional Biochemistry, National Institute of Gastroenterology IRCCS “Saverio de Bellis”, 70013 Castellana Grotte, Italy; maria.notarnicola@irccsdebellis.it (M.N.); valeria.tutino@irccsdebellis.it (V.T.); valentina.denunzio@irccsdebellis.it (V.D.N.); giuliano.pinto@irccsdebellis.it (G.P.)

**Keywords:** IBS-D, low-FODMAP diet, depression, intestinal permeability, inflammation, gut–brain axis

## Abstract

**Introduction:** Alterations of the microbiota–gut–brain axis, including increased intestinal permeability (IP), changes in microbial activity, and immune activation, are central to the pathophysiology of irritable bowel syndrome with diarrhea (IBS-D). The low-fermentable oligo-di-monosaccharides and polyols (FODMAP) diet (LFD) is an established therapy for IBS, yet its systemic effects, particularly in patients with elevated depressive symptoms, remain incompletely characterized. **Methods:** This single-arm pre–post study investigated associations between depressive symptom severity and markers of small IP (s-IP), endotoxin exposure, inflammation, and erythrocyte membrane polyunsaturated fatty acid (PUFA) composition in 43 IBS-D patients undergoing a 12-week personalized LFD. Patients were classified using the Symptom Checklist-90-Revised depression subscale into those with (d+, *n* = 23) and without (d−, *n* = 20) clinically elevated depressive symptoms. **Results:** At baseline, d+ patients exhibited higher s-IP, circulating lipopolysaccharide levels, inflammatory markers, and a more pro-inflammatory PUFA profile. Following LFD, significant improvements in symptoms and several biological parameters were observed in the overall cohort. Greater absolute changes in d+ patients were consistent with their higher baseline values rather than indicating differential responsiveness. Baseline depressive symptoms were not significantly associated with the magnitude of post-intervention changes in IP or inflammatory markers. **Conclusions:** These findings suggest that elevated depressive symptoms identify an IBS-D subgroup characterized by greater baseline biological burden. Results should be interpreted as associative given the single-arm design, absence of a control group, and the concurrent reduction in body weight, which may have influenced the observed changes. Randomized controlled studies are needed to clarify the role of dietary interventions in modulating gut–brain axis-related pathways in IBS-D.

## 1. Introduction

Irritable bowel syndrome (IBS) is one of the most prevalent functional gastrointestinal (GI) disorders affecting approximately 4% of the global population, according to the most recent Rome IV diagnostic criteria [1]. It stands as a paradigmatic biopsychosocial disorder, in which psychological factors actively modulate GI physiology rather than merely co-occurring with it [2]. Clinical evidence now suggests that stress and mood disorders can directly alter intestinal motility, visceral sensitivity, and immune activation via the autonomic and neuroendocrine pathways.

In IBS cohorts, depressive symptoms have been specifically associated with microbiota compositional shifts and compromised intestinal barrier function [3,4], underscoring the bidirectional nature of gut–brain communication. Central to this communication is the intestinal barrier, a dynamic interface that regulates the translocation of nutrients and microbial metabolites [5]. This structure governs luminal antigen translocation and shapes systemic immune tone. When this barrier is disrupted, a state commonly referred to as increased intestinal permeability (IP) or “leaky gut,” lipopolysaccharide (LPS) gains access to the systemic circulation, triggering low-grade mucosal and systemic inflammation. This dysfunction is especially pronounced in IBS patients with the diarrhea-predominant subtype (IBS-D) [6], where its consequences extend beyond the gut to influence cognitive and affective processing [7,8]. Current evidence suggests that these pathways may synergistically contribute to intestinal barrier impairment, a pathological feature increasingly recognized as a pivotal link between the pathophysiology of IBS and mood disturbances [9].

Polyunsaturated fatty acids (PUFAs) represent another modifiable axis within this framework [10]. Indeed, altered PUFA metabolism, specifically omega-3 PUFA (*n*-3) deficiency and a relative predominance of omega-6 (*n*-6) fatty acids (FA), are well-documented features of depressive states [11], and erythrocyte membrane lipid profiles have emerged as stable, biologically integrated biomarkers of both clinical subtype and treatment responsiveness [12]. Recent research by our group has further demonstrated a close relationship between dysregulated PUFA metabolism and impaired barrier function in IBS-D patients with comorbid depression, indicating that markers of erythrocyte membrane PUFA levels and small IP (s-IP) may be potential therapeutic targets [13]. Erythrocyte membrane profiling, in contrast to plasma levels, offers a reliable “snapshot” of long-term nutritional and metabolic status, reflecting the interaction of diet and genetics over time [14].

Patients with IBS and comorbid depressive features report more frequent symptom exacerbation in response to specific foods, suggesting that mood status may amplify dietary sensitivity [15]. The low-fermentable oligosaccharides, disaccharides, monosaccharides, and polyols (FODMAP) diet (LFD) has been demonstrated to be effective in controlling IBS symptoms and supporting barrier restoration [16,17], partly by attenuating microbial fermentation and luminal gas production, thereby stabilizing gut–brain axis homeostasis [18]. Our previous research confirms that LFD is associated with improvements in markers of barrier function and improves PUFA composition in some IBS-D patients [19]. Despite epidemiological evidence linking overall diet quality to depression risk [20,21], few interventional trials have systematically examined the effects of targeted dietary regimens on permeability, dysbiosis, cytokine profiles, or PUFA composition in this population [22,23].

The present single-arm study was therefore designed to investigate associations between depressive symptom severity and baseline measures of s-IP, endotoxemia, inflammatory markers, gut dysbiosis, and erythrocyte PUFA composition. We further examined whether these biological parameters predicted the magnitude of response to a 12-week LFD intervention. Beyond symptom-level outcomes, the study sought to delineate biological signatures that could inform the development of personalized nutritional strategies within the gut–brain axis framework.

## 2. Materials and Methods

### 2.1. Patient Recruitment

Participants were recruited from the Functional Gastrointestinal Disorders Unit of the National Institute of Gastroenterology “S. de Bellis” Research Hospital (Castellana Grotte, Italy) between January and May 2022. The cohort consisted of Italian Caucasians patients diagnosed with IBS-D, according to the Rome IV criteria. Upon enrollment, each subject underwent a thorough physical examination, and baseline urine and serum samples were collected for analysis. To ensure a standardized study population, inclusion was restricted to patients aged 18–65 years with a minimum 12-week history of symptoms, a baseline IBS severity scoring system (IBS-SSS) score > 75, and a fecal pattern consistent with the criteria established by Schmulsson et al. [24].

A rigorous screening protocol was implemented to rule out organic diseases. This included blood panels for liver and thyroid function, C-reactive protein (measured within the previous three months), and a comprehensive stool analysis (triple-sample occult blood, culture, and parasite testing). All patients underwent gastroscopy and colonoscopy to exclude structural abnormalities. Celiac disease was ruled out via tissue transglutaminase and anti-endomysial antibody testing; furthermore, only HLA-DQ2/HLA-DQ8-negative patients were included to exclude celiac disease; however, this does not rule out non-celiac gluten sensitivity. We excluded individuals who had recently followed restrictive diets (e.g., low-FODMAP, gluten-free, or vegan). Additional exclusion criteria comprised: post-infectious IBS, giardiasis, or previous abdominal surgery, systemic comorbidities (metabolic, endocrine, cardiovascular, renal, or hepatic diseases), pregnancy, malignancy, or current febrile states, strenuous physical activity, recent use (within two weeks) of antibiotics, probiotics, antidepressants (including SSRIs), or medications known to influence GI motility or abdominal pain.

All participants provided written informed consent for data collection. The study protocol was approved by the Local Scientific Committee and the Institutional Ethics Committee of the IRCCS Oncological Hospital—John Paul II Cancer Institute, Bari, Italy (No. 274/E.V.). The clinical trial referenced in our manuscript (NCT03423069) was first posted on ClinicalTrials.gov on 30 January 2018. Any reasons for study withdrawal, ranging from adverse events to loss of follow-up, were systematically documented in individual case report forms.

### 2.2. Study Design

The study was structured around three clinical touchpoints. During the baseline visit (visit 1), patients were briefed on the study objectives and provided written informed consent. Initial gastroenterological examinations were performed, while specialized nutritionists evaluated participants’ baseline dietary habits, medical history, and lifestyle. To establish a reliable baseline, patients were instructed to maintain their usual diet for 1 week and to document food intake, physical activity, medications, and stool patterns in a 7-day diary. At visit 2 (dietary assignment), researchers reviewed these diaries to confirm eligibility. Following anthropometric measurements, patients underwent a sugar absorption test (SAT) and provided serum, stool, and urine samples. Each participant then received a personalized dietary plan. Throughout the 12-week intervention, patients continued to log their daily food intake, physical exercise, and GI symptoms in their diaries. The final assessment (visit 3) took place at the end of the 12 weeks. Participants returned their completed symptom and dietary logs. Treatment adherence was rigorously evaluated using the IBS-SSS and the IBS Diet Adherence Report Scale. Finally, all anthropometric and biochemical measurements were repeated to assess the intervention’s impact.

IBS-D patients were subsequently stratified into two subgroups based on the presence (d+) or absence (d−) of depressive symptoms, defined by the Symptom Checklist-90-Revised (SCL-90-R) depression subscale T-score ≥ 63. Patients were enrolled consecutively without randomization, and no blinding was applied to outcome assessors or to depression status classification, which was determined post hoc based on SCL-90-R scores. Accordingly, this study should be considered an exploratory, hypothesis-generating analysis; findings should not be interpreted as confirmatory evidence.

### 2.3. Nutritional Intervention: The Low-FODMAP Diet

The personalized LFD was prescribed at visit 2, following a consultation with the study nutritionists. The primary goal of the LFD is to restrict the intake of highly fermentable short-chain carbohydrates [25]. To ensure nutritional adequacy, Nutrigeo 8.6.0.0 software (Progeo Medical, Monteprandone, Italy) was used to standardize the macronutrient distribution to approximately 50% carbohydrates, 30% fats, and 20% protein. Diet formulation followed the methods described in previous research [18]. Each patient was provided with a comprehensive meal plan, consisting of three main meals and two snacks. To support adherence, an educational leaflet based on the Monash University guidelines was also distributed, detailing permitted and prohibited foods [26]. While fiber intake was kept at physiological levels to maintain bowel regularity, alcohol consumption was discouraged throughout the study period.

### 2.4. Assessment of Nutrient Intake

To evaluate energy intake and expenditure, patients were required to maintain a detailed food diary both at baseline and throughout the 12-week intervention. These records were systematically reviewed by study nutritionists, who documented the type and quantity (in grams) of all food consumed during main meals and snacks, alongside the nature and duration of daily physical activity [17]. Nutritional data were processed using the Progetto Dieta software (v. 2.0, Angelo Manna, Italy), which enabled precise calculation of daily macronutrient distribution (carbohydrates, proteins, and lipids), total dietary fiber, and alcohol consumption. Furthermore, the software provided estimates of total daily energy intake and expenditure, expressed in kilocalories, ensuring that the prescribed diet remained isoenergetic and nutritionally balanced for each participant.

### 2.5. Anthropometric Assessment

Anthropometric parameters, including body weight, height, and waist circumference, were recorded for all participants. Body weight and height were measured using a SECA 700 mechanical column scale and a SECA 220 stadiometer (INTERMED S.r.l., Milan, Italy), respectively. These values were subsequently used to calculate the body mass index (BMI) expressed in kg/m^2^. Additionally, waist circumference was determined using a SECA 201 calibrated tape measure (INTERMED S.r.l., Milan, Italy), ensuring standardized placement to maintain consistency across all evaluation points.

### 2.6. Symptom Profile Assessment

#### 2.6.1. Gastrointestinal Symptomatology

GI symptoms were quantified using the validated IBS-SSS [27]. This multidimensional tool evaluates five key domains: abdominal pain severity, abdominal pain frequency, abdominal distension, dissatisfaction with bowel habits, and the overall impact of symptoms on quality of life. Each domain is assessed using a 100-point visual analog scale, yielding a cumulative score ranging from 0 to 500. Scores were used to categorize disease severity as mild (75–175), moderate (>175–300), or severe (>300), with clinical remission defined by a total score below 75.

#### 2.6.2. Psychological Assessment

Psychological distress was evaluated through the SCL-90-R, a widely recognized self-report instrument for assessing psychopathology in clinical populations [28]. The tool spans nine primary dimensions: somatization, obsessive-compulsive symptoms, interpersonal sensitivity, depression, anxiety, hostility, phobic anxiety, paranoid ideation, and psychoticism. Raw scores for each dimension were converted into standardized T-scores. Consistent with established clinical thresholds, T-scores ≥ 63 were utilized to identify clinically significant symptomatology [29]. Given the established role of psychological factors in functional GI disorders, particular attention was paid to the somatization and depression subscales. These were treated both as categorical variables for group stratification and as continuous variables for correlation analyses to capture the full spectrum of psychological distress.

### 2.7. Biochemical and Molecular Analyses

#### 2.7.1. Intestinal Permeability and Barrier Integrity

Small-IP was evaluated both at baseline and post-intervention via the SAT. Participants’ urinary recovery of lactulose (Lac) and mannitol (Man) was measured to assess paracellular integrity and transcellular pathways, respectively. Urinary concentrations were determined using chromatographic analysis as previously described [30]. The percentages of ingested Lac and Man excreted (%Lac, %Man) were calculated to derive the Lac/Man ratio, with values exceeding 0.03 considered indicative of impaired barrier function [31]. Fecal zonulin, a protein involved in the modulation of tight junctions (TJs) [32], was measured as a supportive marker of intestinal barrier function using ELISA kits (Immunodiagnostik AG, Bensheim, Germany). However, its validity as a quantitative biomarker of intestinal permeability remains debated. Stool samples were processed and stored at −80 °C within 12 h of collection. Serum intestinal FA-binding protein (I-FABP), a marker of enterocyte damage, Ref. [33], was analyzed using ELISA kits from Cloud-Clone Corp (Houston, TX, USA).

#### 2.7.2. Dysbiosis, Translocation, and Inflammatory Signaling

An indirect surrogate marker of fermentative dysbiosis was assessed by measuring urinary indican levels using a colorimetric assay (ABNOVA Corp, Taoyuan City, Taiwan); values exceeding 20 mg/L indicated fermentative dysbiosis. Circulating LPS levels were measured as a proxy of systemic endotoxin exposure using ELISA (Cloud-Clone Corp, TX, USA). The systemic inflammatory response was assessed through a panel of serum cytokines and lipid mediators. Interleukins (IL-6, IL-8, IL-10) and tumor necrosis factor-alpha (TNF-α) were quantified using ELISA kits (ElabScience Biotechnology Inc., Houston, TX, USA).

#### 2.7.3. Erythrocyte Membrane Fatty Acid Profiling

The FA composition of erythrocyte membranes was analyzed to provide a stable indicator of long-term lipid metabolism and inflammatory status. Whole blood collected in EDTA tubes was centrifuged (4000× *g* for 5 min at 6 °C) to isolate mature erythrocytes. The extraction and transesterification of lipids into FA methyl esters (FAMEs) were performed using an automated Robot LNG-R1 system (Lipinutragen-Tecan, Bologna, Italy). The resulting FAMEs were resuspended in n-hexane and analyzed by gas chromatography equipped with a flame ionization detector and a hydrogen gas generator (Thermo Fisher Scientific, Milan, Italy) as described previously [19]. Quantification was based on a 37-component standard FAMEs mix (Supelco, Sigma-Aldrich, Milan, Italy). PUFAs are expressed as the mean percentage of red blood cell membrane composition (% rel).

### 2.8. Statistical Analysis

Statistical analyses were performed using SigmaStat software (v. 4.0, Systat Software Inc., San Jose, CA, USA). Data are expressed as means ± SD. Given the sample size and to avoid assumptions regarding population distribution, non-parametric tests were prioritized where appropriate. Changes from baseline to post-intervention were assessed using paired *t*-tests for normally distributed variables or Wilcoxon signed-rank tests for non-normally distributed data. Correlations between psychological scores and biological markers were analyzed using the Spearman rank correlation coefficient. Multivariable linear regression analyses were performed to explore the predictive value of psychological distress on biological outcomes. The first model (Baseline Permeability) examined whether baseline depression scores predicted the Lac/Man ratio while adjusting for age and BMI. The second model (endotoxin exposure) evaluated baseline LPS levels as the dependent variable, with depression score, Lac/Man ratio, age, and BMI as independent predictors. The third model (Treatment Response) was conducted to determine if baseline depression predicted the clinical response to the LFD. For these analyses, delta values (post-pre) of the Lac/Man ratio and IL-6 were used as dependent variables. Effect sizes (r) for non-parametric tests were calculated as r = Z/√N, and 95% confidence intervals were estimated for the main outcomes (Table S1).

Model fit was assessed using the coefficient of determination (R^2^). For all analyses, a two-tailed *p*-value < 0.05 was considered statistically significant. Given the large number of comparisons performed, no correction for multiple testing was applied; all *p*-values should therefore be interpreted as exploratory and hypothesis-generating. No a priori sample size calculation was performed; post hoc analysis indicated that 43 participants provided >80% power to detect clinically meaningful differences. The study should be regarded as exploratory. Full regression model details, including regression coefficients (β), standard errors, *p*-values, and R^2^ values for all predictors, are reported in Supplementary Table S2. For each variable, normality was assessed automatically by SigmaStat software, which selected paired *t*-tests for normally distributed variables and Wilcoxon signed-rank tests for non-normally distributed data. Given the limited sample size, especially within subgroups, all regression models should be interpreted as exploratory, with caution regarding the stability and generalizability of coefficient estimates.

## 3. Results

### 3.1. Patient Flow, Anthropometrics, and Dietary Adherence

**Figure 1:**
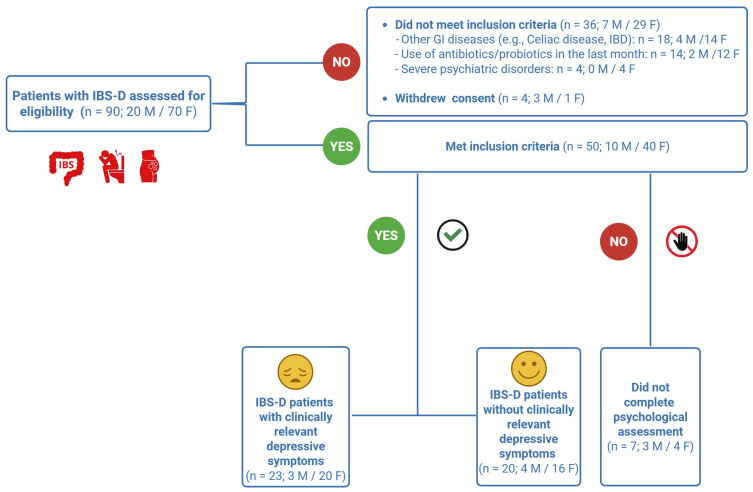
Flowchart of patient recruitment and study inclusion. IBS-D: irritable bowel syndrome with diarrhea; M: male; F: female. GI: gastrointestinal.

The study flow and participant transition throughout the intervention are summarized in Figure 1. Initially, 90 patients with IBS-D (20 males, 70 females) were screened for eligibility. Of these, 40 were excluded: 36 failed to meet the specific inclusion criteria, and 4 withdrew their consent. The remaining 50 patients (10 males, 40 females) were enrolled in the study. Complete psychological assessments were obtained for 43 participants. Based on the SCL-90-R depression subscale, 23 patients were categorized as patients with clinically relevant depressive symptoms [(d+); 3 males, 20 females], while 20 were classified as patients without clinically relevant depressive symptoms [(d−); 4 males, 16 females]. The seven patients (3 males, 4 females) who did not complete the psychological screening were excluded from all subsequent analyses involving psychological variables.

The anthropometric profile of the cohort at baseline and following the 12-week intervention is detailed in Table 1. Significant reductions (*p* < 0.05) in body weight, BMI, and waist circumference were observed across the entire study population. Despite the diet being designed as isoenergetic, these improvements remained consistent across the d+ and d− subgroups. At baseline, the d+ and d− subgroups were comparable regarding age (*p* = 0.26), BMI (*p* = 0.84), and waist circumference (*p* = 0.81). No significant differences were observed between the two groups in terms of IBS-SSS total score (*p* = 0.86) or individual symptom severity. Finally, analysis of the daily food diaries confirmed that dietary compliance was high and comparable between both groups throughout the study period.

### 3.2. Gastrointestinal Symptom Profile

IBS-SSS scores decreased significantly after the intervention in both subgroups, with no difference between d+ and d− patients (Table 2).

### 3.3. Psychological Profile

At baseline, the study population exhibited significant psychological distress, with mean SCL-90-R scores for somatization, depression, and anxiety all exceeding the clinical threshold of 63 (Table 3). These data underscore a substantial burden of both affective and somatic symptoms within our IBS-D cohort.

Stratification by depressive status revealed two distinct psychological phenotypes. Patients in the d+ group showed elevated scores across nearly all SCL-90-R dimensions, suggesting a generalized pattern of psychological distress rather than isolated depressive symptoms. In contrast, the d− subgroup presented a psychological profile that remained consistently below pathological thresholds at study entry. Following the 12-week LFD intervention, a widespread reduction in psychological distress was observed across the entire sample, with most scores returning to the non-clinical range. In the d+ subgroup, improvements were evident across almost all domains. However, it is noteworthy that while their depressive symptoms decreased, they remained slightly above the clinical cut-off (66.39 ± 3.60), suggesting that dietary intervention provided significant but incomplete relief of affective distress. For the d− group, only marginal fluctuations were observed, as their scores were already within the normal range at baseline.

### 3.4. Biomarkers of Intestinal Barrier Function and Integrity

**Figure 2:**
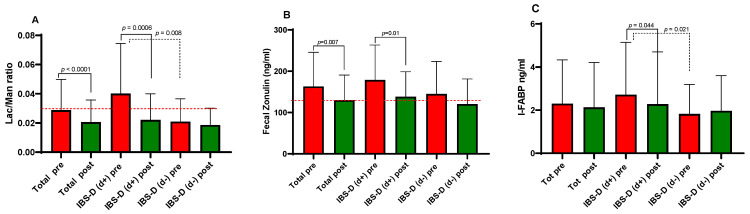
Urinary, fecal and serum biomarkers of intestinal barrier function and integrity in diarrhea-predominant irritable bowel syndrome (IBS-D) patients as a whole group and categorized in d+ and d− subgroups according to depression symptoms at baseline (cutoff score = 63), before (pre) and after (post) 12 weeks of the low FODMAP diet. Lac/Man (%Lac to %Man) ratio (**A**), fecal zonulin (**B**), and intestinal fatty acid-binding protein (I-FABP) (**C**). Data expressed as means ± SD. The Wilcoxon signed-rank test (solid line) was used to compare pre- and post-treatment data. The Mann–Whitney test (dotted line) was used to compare the two subgroups before and after the diet. Differences considered significant at *p* < 0.05. The red dotted line indicates the cutoff value. Individual data points and paired plots are shown in Supplementary Figure S2.

The 12-week LFD intervention led to significant improvements in intestinal barrier function. In the overall IBS-D cohort, both urinary % Lac and % Man recovery decreased significantly (Figure 2A). Specifically, %Lac dropped from 0.38 ± 0.05 vs. 0.26 ± 0.03 (*p* < 0.0001), while %Man decreased from 13.44 ± 0.51 vs. 12.72 ± 0.51 (*p* = 0.028). Consequently, the cumulative Lac/Man ratio showed a marked reduction (0.031 ± 0.004 vs. 0.020 ± 0.002, *p* < 0.0001). A key finding emerged when stratifying the groups: at baseline, the d+ subgroup exhibited a significantly higher Lac/Man ratio than d− patients (0.040 ± 0.007 vs. 0.020 ± 0.003, *p* = 0.0006). Notably, the mean ratio in d+ patients was well above the 0.030 clinical cutoff, indicating overt barrier impairment. Following the LFD, the Lac/Man ratio in the d+ group decreased by approximately 50%, effectively normalizing and reaching levels comparable to those of the d− subgroup. By the end of the study, no significant differences remained between the two cohorts.

Similar trends were observed for fecal zonulin (Figure 2B). In the entire group, zonulin levels fell significantly from 163.10 ± 12.59 ng/mL vs. 129.90 ± 9.23 ng/mL (*p* = 0.007). At study entry, the d+ group showed higher baseline zonulin levels than the d− group (178.9 ± 17.64 ng/mL vs. 145.0 ± 17.51 ng/mL), although this did not reach statistical significance. However, the changes consistent with improved barrier function were noted in the d+ subgroup, in which zonulin levels decreased significantly to 138.3 ± 12.58 ng/mL (*p* = 0.01). In contrast, no significant change was observed in the d− subgroup.

Finally, serum I-FABP levels, a marker of enterocyte damage, mirrored these improvements (Figure 2C). While overall levels remained relatively stable, baseline concentrations were significantly higher in d+ compared to d− patients (2.71 ± 0.50 ng/mL vs. 1.82 ± 0.30 ng/mL, *p* = 0.021). The LFD successfully reduced I-FABP levels only in the patients with clinically relevant depressive symptoms subgroup (2.27 ± 0.50 ng/mL, *p* = 0.044), while the patients without clinically relevant depressive symptoms remained stable. As with the other markers, the initial disparity between subgroups was no longer detectable at the 12-week follow-up.

### 3.5. Biomarkers of Fermentative Dysbiosis (Indican), Endotoxin Exposure, and Inflammation

**Figure 3:**
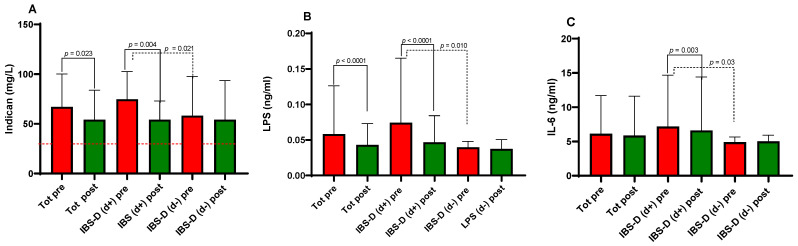
Urinary indican (surrogate marker of fermentative dysbiosis), circulating LPS levels, interpreted as a proxy of endotoxin exposure and the indices of inflammation in diarrhea-predominant irritable bowel syndrome (IBS-D) patients as a whole group and categorized in d+ and d− subgroups according to depression symptoms at baseline (cutoff score 63), before (pre) and after (post) 12 weeks of the low-FODMAP diet. Indican (**A**), lipopolysaccharide (LPS) (**B**), and interleukin-6 (IL-6) (**C**). Data expressed as means ± SD. The Wilcoxon signed-rank test (solid line) was used to compare pre- and post-treatment data. The Mann–Whitney test (dotted line) was used to compare the two subgroups before and after the diet. Differences considered significant at *p* < 0.05. The red dotted line indicates the cutoff value. Individual data points and paired plots are shown in Supplementary Figure S3.

The effects of the LFD on the surrogate marker of fermentative dysbiosis (indican) and systemic markers are summarized in Figure 3. Regardless of psychological status, baseline urinary indican levels in all IBS-D patients exceeded the 20 mg/L threshold, indicating prevalent fermentative dysbiosis. However, the intervention led to a significant overall reduction in indican concentration (67.09 ± 5.24 mg/L, vs. 54.19 ± 4.53 mg/L; *p* = 0.023). When examining the subgroups, we found that d+ patients started with significantly higher indican levels than d− patients (74.78 ± 5.79 mg/L vs. 58.25 ± 8.29 mg/L; *p* = 0.021). The dietary intervention was particularly effective in the d+ subgroup, where levels dropped significantly (*p* = 0.004), whereas the d− subgroup remained largely unchanged. By the end of the 12 weeks, the disparity between the two subgroups had vanished (Figure 3A).

Serum LPS levels, a key marker of circulating LPS levels, interpreted as a proxy of endotoxin exposure, followed a similar pattern (Figure 3B). In the entire cohort, LPS decreased significantly from baseline to the end of the study (0.058 ± 0.01 ng/mL vs. 0.043 ± 0.011 ng/mL; *p* < 0.0001). Again, baseline LPS was notably higher in the d+ group compared to the d− group (*p* = 0.010). Post-LFD, only the d+ patients showed a significant reduction in LPS (*p* < 0.0001), while the d− group showed stable levels. At the conclusion of the study, serum LPS concentrations were comparable between both subgroups.

Regarding systemic inflammation, while overall IL-6 levels remained stable, significant differences were observed between subgroups at baseline (*p* = 0.033). In the d+ subgroup, IL-6 levels decreased significantly after the treatment (7.17 ± 1.56 ng/mL vs. 6.60 ± 1.62 ng/mL, *p* = 0.003), whereas no change was observed in the d− group (Figure 3C). By the end of the study, the two groups showed no significant differences in IL-6 concentrations. Other inflammatory markers, including IL-8, IL-10, and TNF-α, did not change significantly.

### 3.6. Erythrocyte Membrane PUFA Profiles

**Figure 4:**
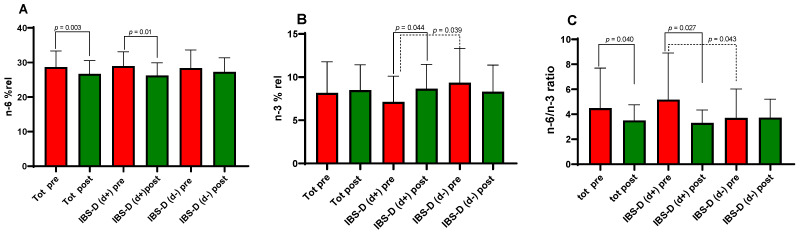
Mean percentage of red blood cell membranes PUFAs (%rel) in diarrhea-predominant irritable bowel syndrome (IBS-D) patients as a whole group and categorized in d+ and d− subgroups according to depression symptoms at baseline (cutoff score 63), before (pre) and after (post) 12 weeks of the low-FODMAP diet. Omega-6 polyunsaturated fatty acids (*n*-6) % rel (**A**), Omega-3 polyunsaturated fatty acids (*n*-3) % rel (**B**), and *n*-6/*n*-3 ratio (**C**). Data expressed as means ± SD. The Wilcoxon signed-rank test (solid line) was used to compare pre- and post-treatment data. The Mann–Whitney test (dotted line) was used to compare the two subgroups before and after the diet. Differences considered significant at *p* < 0.05.

The impact of the LFD on the composition of PUFAs in red blood cell membranes is illustrated in Figure 4. In the overall IBS-D population, a significant reduction in n-6 PUFAs levels was observed following the dietary intervention (28.68 ± 0.70 vs. 26.71 vs. 0.58, *p* = 0.003). This trend was particularly evident in the d+ subgroup, where *n*-6 levels dropped from 28.94 ± 0.86 to 26.22 ± 0.76 (*p* = 0.01; Figure 4A).

Regarding *n*-3 PUFAs, the d+ group had significantly lower levels at baseline than the d− group (*p* = 0.039). However, the 12-week LFD intervention successfully reversed this deficit in the d+ subgroup, leading to a significant increase in *n*-3 concentrations (7.13 ± 0.62 vs. 8.65 ± 0.59, *p* = 0.044; Figure 4B). The most striking changes were observed in the *n*-6/*n*-3 ratio, a systemic indicator of pro-inflammatory status. In the overall cohort, this ratio decreased significantly after LFD (4.48 ± 0.48 vs. 3.50 ± 0.19; *p* = 0.040). Subgroup analysis revealed that d+ patients had a significantly higher (more pro-inflammatory) baseline ratio than d− patients (*p* = 0.043). Following treatment, the d+ group showed a profound reduction in this ratio (5.17 ± 0.77 vs. 3.30 ± 0.21; *p* = 0.027; Figure 4C), effectively reaching levels similar to those of the d− group. In contrast, no significant changes in any PUFAs parameters were noted in the d− subgroup. By the end of the 12-week study, the erythrocyte membrane lipid profiles were statistically indistinguishable between the two subgroups.

### 3.7. Correlations and Multivariate Regression Analyses

In the d+ subgroup, indican was positively correlated with LPS (r = 0.465, *p* = 0.025) and I-FABP (r = 0.405, *p* = 0.049). No significant correlations were observed in the d− subgroup.

To formally test whether the magnitude of change differed between subgroups, a group × time interaction analysis was performed by comparing pre-to-post delta scores (Δ) between d+ and d− patients using the Mann–Whitney U test. Significant interactions were observed for the Lac/Man ratio (U = 104.0, *p* = 0.002, r = 0.47), LPS (U = 118.5, *p* = 0.007, r = 0.41), IL-6 (U = 121.5, *p* = 0.008, r = 0.40), I-FABP (U = 145.5, *p* = 0.041, r = 0.31), indican (U = 148.5, *p* = 0.048, r = 0.30), *n*-3 PUFAs (U = 329.5, *p* = 0.016, r = 0.37), and the *n*-6/*n*-3 ratio (U = 107.0, *p* = 0.003, r = 0.46), indicating that the d+ subgroup showed a significantly greater response to the dietary intervention for these outcomes. No significant group × time interaction was detected for fecal zonulin (*p* = 0.43) or *n*-6 PUFAs (*p* = 0.21).

Multivariate linear regression was employed to further explore the association between baseline depressive symptoms and markers of intestinal barrier dysfunction and systemic inflammation. At baseline, depressive symptom severity was significantly associated with intestinal permeability (Lac/Man ratio; β = 0.000516, *p* = 0.004; R^2^ = 0.198) and circulating LPS levels (β = 0.00148, *p* = 0.016; R^2^ = 0.253), independent of age and BMI. However, baseline depressive symptom severity did not significantly predict changes in IP following the intervention (ΔLac/Man; β = 0.0000886, *p* = 0.388). Instead, baseline Lac/Man values were the only significant predictor of change (β = 0.422, *p* < 0.001; R^2^ = 0.478). Similarly, baseline depressive symptoms were not significantly associated with changes in IL-6 levels (ΔIL-6; β = 0.0141, *p* = 0.070), and the overall model was not statistically significant (R^2^ = 0.081, *p* = 0.184). Full regression results are reported in Supplementary Table S2.

Spearman correlation between changes in IBS-SSS total score (ΔIBS-SSS) and biological outcomes showed no significant correlations in the overall cohort or d+ subgroup. In contrast, in the d− subgroup, ΔIBS-SSS correlated positively with ΔLac/Man ratio (r = 0.565, *p* = 0.009), indicating that greater reductions in IP were associated with greater symptomatic improvement.

## 4. Discussion

This study examined the relationship between depressive symptom severity and biological markers related to intestinal barrier function, endotoxin exposure, inflammation, and PUFA composition in IBS-D patients undergoing a 12-week low-FODMAP dietary intervention. The main finding is that patients with clinically relevant depressive symptoms (d+) exhibited a higher baseline biological burden across multiple domains, including increased IP, endotoxin exposure, and a more pro-inflammatory lipid profile.

After 12 weeks of LFD, both groups demonstrated measurable biological improvements. Formal group × time interaction analyses confirmed that the d+ subgroup showed a significantly greater response compared to d− for most primary biological outcomes, while no significant differential response was observed for fecal zonulin or *n*-6 PUFAs. These findings suggest that the larger absolute changes observed in d+ patients are at least partially attributable to their higher baseline burden, although formal group × time interaction analyses confirmed significantly greater changes in d+ for most biological outcomes.

First and foremost, however, it should be noted that in patients with IBS, physical symptoms and psychological distress often overlap, making it difficult to distinguish between them. Since the SCL-90-R measures psychological distress, the greater symptom burden in the d+ group may also reflect somatization processes. The improvement following the LFD diet could therefore indicate an overall reduction in psychophysical distress, not just in intestinal symptoms.

However, given the single-arm pre–post design and the absence of a control group, these changes cannot be attributed specifically to the LFD. Non-specific effects, regression to the mean, or natural symptom variability may have contributed to the observed improvements.

A key confounder is the significant reduction in body weight, BMI, and waist circumference across the cohort despite the isoenergetic design. The causes are unclear and may include underreported intake, behavioral changes, or increased physical activity.

Weight loss itself is a known independent modulator of many outcomes assessed (e.g., IL-6, lipid profile including *n*-6/*n*-3 PUFA ratio, and intestinal permeability). Therefore, the relative contribution of FODMAP restriction versus weight loss to the observed biological changes cannot be determined. This limitation should be considered when interpreting subsequent findings.

Overall, depressive symptoms appear to identify a biologically distinct IBS-D subgroup with greater gut–brain axis dysregulation, characterized by a higher baseline biological burden, without evidence of greater response after adjustment to targeted nutritional interventions.

Depression severity was independently associated with baseline intestinal permeability (R^2^ = 0.198). Mechanisms that involve dysregulation of the HPA axis and autonomic tone [34] have been proposed as factors promoting epithelial permeability, microbial translocation, and low-grade inflammation, which may partly explain the current findings. Elevated LPS, interpreted here as an indirect estimate of endotoxin exposure rather than direct evidence of bacterial translocation, alongside indican and I-FABP in d+ patients, are consistent with greater systemic gut barrier dysfunction extending beyond the intestinal lumen [35].

Following LFD, reductions in the Lac/Man ratio, zonulin, and I-FABP were observed across the cohort, with formal confirmation that the reductions in the Lac/Man ratio and I-FABP were significantly greater in d+, while zonulin did not show a significant difference, which is biologically plausible. By reducing fermentable carbohydrate intake, LFD likely limits microbial fermentation and luminal metabolites that impair epithelial TJs [36,37], thereby improving barrier function, although it should be noted that fecal zonulin, used here as a supportive marker, has limited specificity as a quantitative index of IP. These effects may extend beyond the intestine, contributing to reduced levels of the surrogate marker of fermentative dysbiosis (indican), lower circulating LPS levels, suggestive of reduced endotoxin exposure, and inflammation [38,39], and ultimately modulating gut–brain axis signaling.

In any case, LFD may represent not only a symptomatic treatment for IBS-D but also an intervention capable of modulating biological pathways involved in gut–brain communication [40]. In d+ patients, the decrease in indican and LPS levels is consistent with the possibility of changes in fermentative processes and reduced endotoxemia, although causal attribution is not possible given the single-arm design. Correlations among indican, LPS, and I-FABP further support this integrated pathway [41,42]. Patients with clinically relevant depressive symptoms showed higher baseline IL-6 levels, which significantly decreased after dietary intervention, supporting the hypothesis that improved barrier integrity is associated with lower levels of endotoxin-related markers. Given the link between inflammation and mood regulation [43], these changes may have broader psychobiological implications in IBS. Notably, d+ patients exhibited an elevated *n*-6/*n*-3 PUFA ratio, consistent with a pro-inflammatory state described in major depressive disorder [44]. Its normalization following LFD suggests a potential role of *n*-3 FA in maintaining barrier integrity and modulating inflammation, although this interpretation remains speculative in the absence of direct mechanistic evidence from this study. As erythrocyte membrane composition reflects long-term exposure, these findings strengthen biological plausibility, although PUFA changes may also reflect reduced systemic inflammation rather than directly contributing to barrier repair.

Interestingly, improvements in biological parameters were accompanied by significant reductions in psychological distress, with SCL-90-R scores improving across the sample after LFD, indicating parallel improvements in psychological distress [45].

Notably, d+ patients remained above the clinical threshold for depression at 12 weeks, suggesting that dietary intervention alone may be insufficient to resolve elevated depressive symptoms; the potential added value of longer-term adherence or combined psychological/pharmacological treatment warrants further investigation.

A bidirectional relationship is suggested, whereby barrier improvement may reduce neuroimmune signaling to mood circuits, while symptom relief may lessen psychological burden; however, this cannot be tested in a single-arm design.

Importantly, depressive symptom severity was associated with baseline biological alterations but did not independently predict the magnitude of post-intervention changes; formal group × time interaction analyses, rather than simple comparisons of within-group significance, were therefore used to assess differential responsiveness.

The correlation analysis revealed a subgroup-specific pattern: while no significant associations between biological and symptomatic changes were observed in the total cohort or in the d+ subgroup, a significant coupling between IP normalization and symptom improvement was found exclusively in d− patients (r = 0.565, *p* = 0.009). This dissociation suggests that in patients with elevated depressive symptoms, symptom perception may be modulated by additional neurobiological or psychological mechanisms beyond gut barrier dysfunction, consistent with current models of the gut–brain axis in IBS.

Several limitations should be acknowledged. All participants were Italian Caucasians recruited from a single clinical center. While the single-center design ensures protocol consistency, it limits the generalizability of the results to populations with different ethnic, cultural, and health backgrounds, particularly given the influence of dietary habits and gut microbiota on IBS symptoms. Furthermore, the stringent exclusion criteria further reduce the study’s representativeness of actual IBS populations.

The sample size, while adequate for primary outcomes, limited the detection of small effects and subgroup analyses. Baseline dietary habits, including habitual FODMAP intake, fiber consumption, and omega-3 intake, were not systematically collected and could not be compared between subgroups. It is therefore possible that pre-existing dietary differences partially contributed to the observed baseline differences in PUFA profiles and IP between d+ and d− patients.

Additionally, the lack of a randomized control group precludes attributing improvements to the LFD, as they may reflect natural symptom variability, non-specific dietary effects, or time-related changes.

Another limitation of this study is that dietary adherence was assessed primarily through self-reported food diaries, which may be prone to recall bias. However, to enhance the reliability of these data, a specialized nutritionist provided thorough instructions and reviewed the diaries at each visit. Moreover, the significant changes observed in objective biomarkers, such as the increase in *n*-3 PUFAs and the reduction in the *n*-6/*n*-3 ratio, provide indirect biological confirmation of the participants’ adherence to the LFD intervention.

Furthermore, the stratification into d+ and d− subgroups was conducted post hoc, and the exclusion of seven patients who did not complete the psychological assessment could represent a potential selection bias. However, a sensitivity analysis comparing baseline GI symptoms and main biomarkers between these excluded subjects and the included cohort revealed no significant differences, suggesting that their exclusion is unlikely to have fundamentally altered the biological findings.

Depressive symptoms were assessed dimensionally (SCL-90-R) without structured DSM-5 diagnostic interviews, limiting diagnostic specificity given the heterogeneity of depression in functional GI disorders. Accordingly, subgroup differences should be interpreted as characterizing patients with differing levels of depressive symptom burden, not as comparisons between diagnostically confirmed depressive disorder and its absence. Detailed sociodemographic variables such as occupational category, marital status, living environment, and income level were not collected, which limits the ability to account for broader psychosocial determinants of symptom burden and treatment response. In addition, the exclusion of patients treated with antidepressants, while necessary to avoid confounding effects on biological and psychological outcomes, may further limit the generalizability of our results, as these medications are frequently prescribed in clinical practice for IBS-D management.

Fecal zonulin was used as a supportive marker; however, its analytical specificity and its role as a proxy for IP remain controversial, which should be considered when interpreting these findings. Plasma LPS measurements based on ELISA are subject to preanalytical and analytical variability; therefore, these values should be interpreted as indirect estimates of endotoxin exposure rather than direct evidence of bacterial translocation.

Finally, the lack of metagenomic and targeted metabolomic analyses limited detailed characterization of microbial changes underlying dysbiosis. The post hoc nature of the subgroup stratification, combined with the absence of blinding, increases the risk of detection bias and limits the interpretability of subgroup-specific findings. This analysis should therefore be considered exploratory and hypothesis-generating rather than confirmatory.

Future studies integrating microbiome sequencing, metabolomics, and longitudinal psychological assessment may help clarify interactions among diet, barrier function, and psychological factors. The potential additive benefit of *n*-3 supplementation in patients with persistently elevated *n*-6/*n*-3 ratios after LFD warrants further investigation. Longer follow-up is needed to assess the durability of barrier restoration and whether sustained dietary adherence yields clinically meaningful improvements in elevated depressive symptoms.

Regression analyses should be interpreted cautiously due to the small sample size, partial violations of model assumptions, and limited statistical power. In the subgroups (*n* = 23 and *n* = 20), the observations-to-predictors ratio approaches the minimum recommended threshold, increasing the risk of overfitting and reducing the stability and generalizability of coefficient estimates. Accordingly, these models are exploratory and should not be used for inferential conclusions about independent predictors of biological outcomes.

The unexpected reduction in body weight observed despite the isoenergetic design represents a significant uncontrolled confounding factor. Weight loss per se is associated with reductions in systemic inflammation, improved lipid profiles, and changes in IP. Without a weight-stable control group, it is not possible to determine the independent contribution of FODMAP restriction versus weight loss to the biological changes observed. This limitation applies particularly to outcomes such as IL-6, the *n*-6/*n*-3 PUFA ratio, and IP markers, all of which are sensitive to changes in adiposity and energy balance independently of dietary composition.

Clinically, depressive symptoms in IBS-D are associated with a distinct biological profile characterized by barrier dysfunction, elevated circulating LPS levels consistent with increased endotoxin exposure, and pro-inflammatory lipid composition, which improves with LFD. Integrating psychological screening into nutritional management may help identify patients who require targeted gut–brain axis interventions, though confirmation from adequately powered randomized trials is needed before broad implementation.

## 5. Conclusions

This study demonstrates that depressive symptoms identify a distinct IBS-D subgroup characterized by increased IP, endotoxemia, low-grade inflammation, altered fermentative activity, and a pro-inflammatory PUFA profile. A 12-week LFD was associated with changes in several biological parameters in both groups. However, the concurrent and unexpected reduction in body weight observed across the cohort, despite the isoenergetic dietary design, represents a significant confounding factor that prevents firm attribution of the biological changes to FODMAP restriction per se. Baseline depressive severity did not predict post-LFD biological changes, suggesting diet efficacy irrespective of psychological status. These findings support the incorporation of standardized psychological screening into routine IBS-D assessment. Tools such as the SCL-90-R depression subscale can help identify patients at higher risk of barrier dysfunction and LPS translocation who may benefit from early dietary intervention. In this context, LFD may act not only on symptoms but also may be linked to changes in gut–brain axis-related markers with measurable biological effects.

However, these results are preliminary. Multicenter randomized controlled trials integrating microbiome profiling, structured psychiatric assessment, and active dietary comparators are necessary to establish causality, identify responder profiles, and evaluate long-term effects.

## Figures and Tables

**Table 1:** Descriptive statistics of anthropometric data before (pre) and after (post) 12 weeks of LFD in the whole group and in subgroups of IBS-D patients according to the presence of d+ or absence of d− of depressive symptoms.

	Total Pre (*n* = 43)	Total Post (*n* = 43)	*p*	d+ Pre (*n* = 23)	d+ Post (*n* = 23)	*p*	d− Pre (*n* = 20)	d− Post (*n* = 20)	*p*
**Weight (kg)**	67.4 ± 12.46	63.49 ± 12.05	<0.0001	67.33 ± 11.52	63.52 ± 11.63	<0.0001	67.47 ± 13.76	63.46 ± 12.82	<0.0001
**Height (m)**	1.623 ± 0.08	1.623 ± 0.08	n.s.	1.62 ± 0.08	1.62 ± 0.08	n.s.	1.624 ± 0.09	1.624 ± 0.09	n.s.
**BMI (kg/m^2^)**	25.58 ± 4.40	24.11 ± 4.34	<0.0001	25.64 ± 4.45	24.21 ± 4.60	<0.0001	25.5 ± 4.46	23.99 ± 4.13	<0.0001
**Waist circ. (cm)**	80.27 ± 12.19	77.28 ± 11.12	<0.0001	80.81 ± 12.22	77.96 ± 11.10	0.0003	79.64 ± 12.43	76.51 ± 11.39	<0.0001

LFD = low fermentable oligosaccharides, disaccharides, monosaccharides, and polyols diet. d+ = patients with clinically relevant depressive symptoms. d− = patients without clinically relevant depressive symptoms. n.s. = not significant. BMI = body mass index. circ. = circumference. Data presented as the means ± SD. Data from before and after treatment were compared using the Wilcoxon signed-rank test. The two subgroups were compared both before and after the diet using the Mann–Whitney test. Differences considered significant at *p* < 0.05. Statistical analysis (Mann–Whitney U test) revealed no significant differences (*p* > 0.05) between d+ and d− groups for any of the baseline parameters listed in this table.

**Table 2:** Descriptive statistics of IBS-SSS scores before (pre) and after (post) 12 weeks of LFD in the overall sample and in IBS-D subgroups according to the presence (d+) or absence (d−) of depressive symptoms.

	Total Pre (*n* = 43)	Total Post (*n* = 43)	*p*	d+ Pre (*n* = 23)	d+ Post (*n* = 23)	*p*	d− Pre (*n* = 20)	d− Post (*n* = 20)	*p*
**Pain severity**	47.33 ± 23.46	21.70 ± 22.04	<0.0001	48.04 ± 23.19	23.48 ± 23.33	<0.0001	46.50 ± 24.34	19.65 ± 20.86	0.0004
**Pain frequency**	47.21 ± 29.30	21.67 ± 25.50	<0.0001	50.00 ± 29.54	20.52 ± 24.70	<0.0001	44.00 ± 29.45	23.00 ± 26.97	0.0009
**Distension**	56.19 ± 22.78	26.35 ± 23.06	<0.0001	54.26 ± 18.67	28.00 ± 26.36	0.0002	58.40 ± 27.10	24.45 ± 19.08	0.0002
**Bowel dissatisfaction**	64.56 ± 23.97	36.60 ± 24.53	<0.0001	64.22 ± 25.57	37.48 ± 26.28	0.0025	64.95 ± 22.64	35.60 ± 23.00	0.0001
**QoL interference**	56.65 ± 22.66	33.40 ± 26.79	<0.0001	60.52 ± 20.88	36.43 ± 29.12	0.0002	52.50 ± 24.31	29.90 ± 24.10	0.0044
**IBS-SSS total**	271.9 ± 81.85	139.7 ± 98.90	<0.0001	277.0 ± 80.87	145.9 ± 116.4	<0.0001	266.1 ± 84.68	132.60 ± 76.31	<0.0001

IBS-SSS = irritable bowel syndrome severity scoring system. LFD = low fermentable oligosaccharides, disaccharides, monosaccharides, and polyols diet. IBS-D = irritable bowel syndrome with diarrhea. d+ = patients with clinically relevant depressive symptoms. d− = patients without clinically relevant depressive symptoms. QoL = Quality of life. Data presented as the means ± SD. Data from before and after treatment were compared using the Wilcoxon signed-rank test. The two subgroups were compared both before and after the diet using the Mann–Whitney test. Differences considered significant at *p* < 0.05. Statistical analysis (Mann–Whitney U test) revealed no significant differences (*p* > 0.05) between d+ and d− groups for any of the baseline parameters listed in this table. Individual data points and paired plots are shown in Supplementary Figure S1.

**Table 3:** Descriptive statistics of SCL-90 R scores before (pre) and after (post) 12 weeks of LFD in the overall sample and in IBS-D subgroups according to the presence (d+) or absence (d−) of depressive symptoms.

	Total Pre (*n* = 43)	Total Post (*n* = 43)	*p*	d+ Pre (*n* = 23)	d+ Post (*n* = 23)	*p*	d− Pre (*n* = 20)	d− Post (*n* = 20)	*p*
**Somatization**	68.05 ± 16.09	57.14 ± 16.61	<0.0001	74.48 ± 14.20	63.91 ± 18.92	0.0003	60.65 ± 15.21	49.35 ± 8.70	0.0005
**Obsession-compulsion**	62.00 ± 18.61	55.95 ± 16.56	0.0017	73.57 ± 17.14	63.83 ± 16.93	0.0012	48.70 ± 8.78	46.90 ± 10.62	0.3161
**Interpersonal Sensitivity**	58.00 ± 16.59	49.30 ± 7.50	<0.0001	67.52 ± 16.65	52.91 ± 7.26	<0.0001	47.05 ± 7.73	45.15 ± 5.41	0.3445
**Depression**	66.09 ± 18.73	57.21 ± 16.96	0.0002	78.70 ± 16.16	66.39 ± 17.25	0.0016	51.60 ± 7.73	46.65 ± 8.46	0.0276
**Anxiety**	64.09 ± 18.98	51.58 ± 14.97	<0.0001	75.30 ± 17.43	58.57 ± 16.65	0.0002	51.20 ± 10.83	43.55 ± 6.92	0.0139
**Hostility**	57.35 ± 11.96	50.58 ± 10.14	<0.0001	62.13 ± 12.53	55.09 ± 11.46	0.0029	51.85 ± 8.65	45.40 ± 4.73	0.0041
**Phobic Anxiety**	57.53 ± 19.41	49.84 ± 8.45	0.0117	63.48 ± 22.72	52.30 ± 10.32	0.0343	50.70 ± 11.97	47.00 ± 4.33	0.2734
**Paranoid Ideation**	56.81 ± 14.85	51.30 ± 12.40	0.0154	63.04 ± 15.51	56.39 ± 13.79	0.1032	49.65 ± 10.39	45.45 ± 7.26	0.0630
**Psychoticism**	64.37 ± 22.03	50.58 ± 10.14	<0.0001	74.78 ± 19.11	55.17 ± 11.30	<0.0001	52.40 ± 19.18	45.30 ± 4.76	0.1563

SCL-90-R = Symptom Checklist-90-Revised. LFD = low fermentable oligosaccharides, disaccharides, monosaccharides, and polyols diet. IBS-D = irritable bowel syndrome with diarrhea. d+ = patients with clinically relevant depressive symptoms. d− = patients without clinically relevant depressive symptoms. Data presented as the means ± SD. Data from before and after treatment were compared using the Wilcoxon signed-rank test. The two subgroups were compared both before and after the diet using the Mann–Whitney test. Differences considered significant at *p* < 0.05.

## Data Availability

The datasets used and/or analyzed during the current study are available at Figshare. https://doi.org/10.6084/m9.figshare.31869496 (accessed on 29 April 2026).

## Supplementary Materials

The following supporting information can be downloaded at https://www.mdpi.com/article/10.3390/nu18091473/s1. Figure S1: Individual data points and paired plots for IBS-SSS scores. Figure S2: Individual data points and paired plots for biomarkers of intestinal barrier function and integrity. Figure S3: Individual data points and paired plots for urinary indican, circulating LPS levels, and IL-6. Table S1: Confidence intervals for the main outcomes. Table S2: Full regression model details including regression coefficients (β), standard errors, *p*-values, and R2 values for all predictors.

## Abbreviations

The following abbreviations are used in this manuscript:

BMI	Body Mass Index
DSM-5	Diagnostic and Statistical Manual of Mental Disorders, 5th Edition
EDTA	Ethylenediaminetetraacetic acid
FA	Fatty Acid
FAMEs	Fatty Acid Methyl Esters
FODMAP	Fermentable Oligosaccharides, Disaccharides, Monosaccharides, and Polyols
GI	Gastrointestinal
HLA	Human Leukocyte Antigen
HPA	Hypothalamic–Pituitary–Adrenal (axis)
I-FABP	Intestinal Fatty Acid-Binding Protein
IBS	Irritable Bowel Syndrome
IBS-D	Irritable Bowel Syndrome with Diarrhea
IBS-SSS	IBS Severity Scoring System
IL-6, IL-8, IL-10	Interleukin-6, Interleukin-8, Interleukin-10
IP	Intestinal Permeability
Lac	Lactulose
LFD	Low-FODMAP Diet
LPS	Lipopolysaccharide
Man	Mannitol
*n*-3	Omega-3 polyunsaturated fatty acids
*n*-6	Omega-6 polyunsaturated fatty acids
PUFA	Polyunsaturated Fatty Acid(s)
QoL	Quality of Life
s-IP	Small Intestinal Permeability
SAT	Sugar Absorption Test
SCL-90-R	Symptom Checklist-90-Revised
SEM	Standard Error of the Mean
TJs	Tight Junctions
TNF-α	Tumor Necrosis Factor-alpha
