# Supplementary material for: Elevated Depressive Symptoms Shape Gut Barrier Integrity, LPS Translocation, and PUFA Composition in IBS-D: Evidence from a Low-FODMAP Dietary Intervention
Source: Nutrients. 2026 May 5;18(9):1473. doi: 10.3390/nu18091473 (PMC13164782; doi:10.3390/nu18091473)
Supplement: Supplementary file 1 [file nutrients-18-01473-s001.zip › Figure S3.pdf]

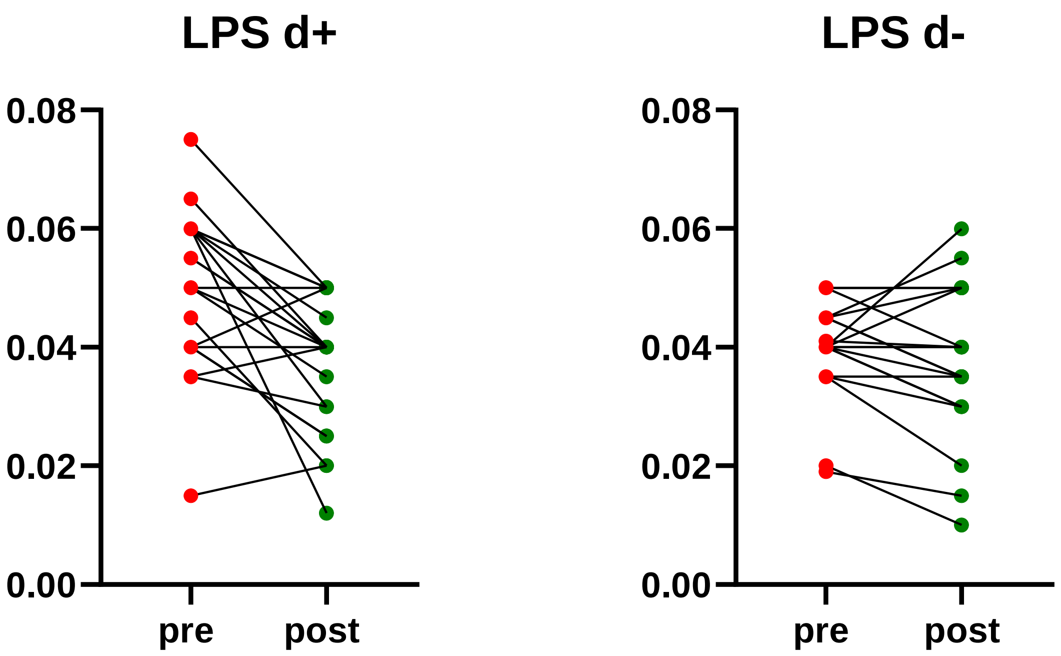

Figure S3. Individual paired changes in LPS before and after the low-FODMAP diet in IBS-D patients with and without clinically relevant depressive symptoms. Individual data points are shown, with lines connecting pre- and post-intervention values.
