# Supplementary material for: Elevated Depressive Symptoms Shape Gut Barrier Integrity, LPS Translocation, and PUFA Composition in IBS-D: Evidence from a Low-FODMAP Dietary Intervention
Source: Nutrients. 2026 May 5;18(9):1473. doi: 10.3390/nu18091473 (PMC13164782; doi:10.3390/nu18091473)
Supplement: Supplementary file 1 [file nutrients-18-01473-s001.zip › Table S1.pdf]

Table S1. Effect sizes and 95% confidence intervals for main outcomes following the 12-week low-FODMAP diet

Outcome	Group	Δ (Post–Pre)	Effect Size (r)	95% CI Of Δ	p
**IBS-SSS total**	Total (n=43)	-135	0.67	-162.7 to -101.7	<0.0001
**IBS-SSS total**	d+ (n=23)	-135	0.92	-173.9 to -88.36	<0.0001
**IBS-SSS total**	d− (n=20)	-160	0.99	-181.0 to -85.88	<0.0001
**Lac/Man ratio**	Total (n=43)	-0.005	0.67	-0.01 to -0.005	<0.0001
**Lac/Man ratio**	d+ (n=23)	-0.010	0.92	-0.003 to -0.008	<0.0001
**Lac/Man ratio**	d− (n=20)	-0.002	0.21	-0.007 to -0.002	0.344
**Fecal zonulin**	Total (n=43)	-20.0	0.41	-55.07 to -11.39	0.0074
**Serum I-FABP**	Total (n=43)	-0.2	0.13	-0.43 to 0.09	0.384
**Indican**	Total (n=43)	-10.0	0.35	-22.33 to -3.48	0.0209
**LPS**	Total (n=43)	-0.010	0.67	-0.03 to 0.0008	<0.0001
**IL-6**	Total (n=43)	0.000	0.27	-0.51 to 0.01	0.0795
**n-6 PUFA (%)**	Total (n=43)	-1.530	0.45	-3.32 to 0.62	0.0030
**n-3 PUFA (%)**	Total (n=43)	+0.64	0.13	-0.77 to 1.43	0.412
**n-6/n-3 ratio**	Total (n=43)	-0.390	0.31	-1.90 to -0.06	0.0401

IBS-SSS = Irritable Bowel Syndrome Severity Scoring System; Lac/Man = lactulose/mannitol; I-FABP = intestinal fatty acid-binding protein; LPS = lipopolysaccharide; IL-6 = interleukin-6; n-3 = Omega-3 polyunsaturated fatty acids; n-6 = Omega-6 polyunsaturated fatty acids; PUFA = polyunsaturated fatty acids. Effect sizes (r) were calculated as $Z/\sqrt{N}$, where Z was derived from the exact two-tailed P value using the inverse normal distribution. Ninety-five percent confidence intervals (95% CI) refer to the mean change (Δ) between post- and pre-intervention values, calculated from the distribution of paired differences.
