# Supplementary material for: Elevated Depressive Symptoms Shape Gut Barrier Integrity, LPS Translocation, and PUFA Composition in IBS-D: Evidence from a Low-FODMAP Dietary Intervention
Source: Nutrients. 2026 May 5;18(9):1473. doi: 10.3390/nu18091473 (PMC13164782; doi:10.3390/nu18091473)
Supplement: Supplementary file 1 [file nutrients-18-01473-s001.zip › Table S2.pdf]

Table S2. Multivariable regression analyses exploring the association between depressive symptoms and biological outcomes

Dependent Variable	Predictor	Β Coefficient	P-Value	Model R^2	Model P-Value
**Lac/Man ratio (baseline)**	Depression score	0.000516	0.004	0.198	0.033
**LPS (baseline)**	Depression score	0.00148	0.016	0.253	0.023
**ΔLac/Man**	Depression score	0.0000886	0.388	0.478	<0.001
**ΔLac/Man**	Lac/Man baseline	0.422	<0.001		
**ΔIL-6**	Depression score	0.0141	0.070	0.081	0.184

Lac/Man: lactulose/mannitol; LPS: lipopolysaccharide; IL-6: interleukin-6. Regression models were adjusted for selected covariates as described in the Methods section. Due to the exploratory nature of the study and the limited sample size, regression coefficients (β) are not available for all predictors and results should be interpreted with caution.
